# How Everything Is Connected to Everything Else – Population‐Specific Connections between Adaptive Evolution, Disease Susceptibility, and Drug Responsiveness

**DOI:** 10.1002/ggn2.202500018

**Published:** 2025-09-10

**Authors:** Ji Tang, Hao Zhu

**Affiliations:** ^1^ Center for Genetic Epidemiology Department of Population and Public Health Sciences Keck School of Medicine University of Southern California Los Angeles CA 90033 USA; ^2^ Bioinformatics Section School of Basic Medical Sciences Southern Medical University Guangzhou 510515 China

**Keywords:** adaptive evolution, disease susceptibility, drug responsiveness, favored mutations, human populations

## Abstract

The genome is like a kaleidoscope through which researchers have obtained varied findings, including favored mutations, disease susceptibility sites, and drug‐responsive sites. Whether these findings have inherent connections is a question deserving investigation. Favored mutations enable humans to adapt to changing environments and lifestyles; however, the adaptation may come with some costs. This is because a favored mutation can change the frequency of varied neutral nucleotides across a large genomic region, and a favored mutation may become disfavored as environments and lifestyles change further. These are the best‐known classes of connections whose causes and consequences have been understood. However, many favored mutations remain unidentified. Using a deep learning network (*DeepFavored*) that integrates statistical tests and is trained on large datasets, favored mutations are recently identified in 17 human populations. The analyses of the results, in conjunction with genome‐wide association study (GWAS) data, suggest that the connection between adaptive evolution, disease susceptibility, and drug responsiveness (referred to as a trade‐off) is extensive and highly population‐specific. The analyses, along with other emerging evidence, suggest that there are other types of connections. In this commentary, these issues are discussed from both retrospective and prospective views, including current challenges and future directions.

## Graphical abstract

Existing studies revealed extensive, population‐specific connections between adaptive evolution, disease susceptibility, and drug responsiveness. This commentary revisits and expands upon these findings to explore the underlying mechanisms. Furthermore, it proposes leveraging key variables in adaptive evolution events to help identify causal mutations and interpret genotype‐phenotype relationships in genome‐wide association studies. Finally, it outlines common limitations in existing studies, presents solutions, and suggests future directions toward uncovering the full scale of the connections.

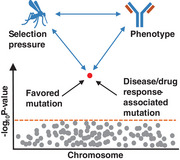

## 1 Introduction

The favored (adaptive) mutations generated in the human genome during and after migrations from Africa to the rest of the world have attracted increasing attention.^[^ ^1^, ^2^ ^]^ They have been generated in response to new habitats and new lifestyles. Thus, different habitats (including changed altitude, temperature, and geographical environments) and lifestyles (including living in large aggregations and on agriculture) have exerted distinct influences on adaptive mutations.^[^ ^1^, ^3^ ^]^ Adaptive mutations have been identified in all human populations and varied genomic elements, but several questions remain unclear, especially a) the scale, b) the onset time, and c) the consequences. It is widely acknowledged that favored mutations detected by statistical tests are greatly underestimated.^[^ ^4^, ^5^ ^]^ This continually drives researchers to explore new methods.^[^ ^1^ ^]^ A promising approach is deep learning networks that integrate statistical tests and big‐data‐based training, which are more powerful than any single test and have the potential to identify multiple kinds of mutations simultaneously.^[^ ^6^ ^]^ However, little is known about questions (b) and (c). The consequences include potential connections between adaptive mutations, the subsequent changes of sequences and nucleotide frequencies, and the resulting genotype‐phenotype relations. The understanding of these consequences remains at an early stage.

A key consequence is the potential connection between adaptive mutations and disease susceptibility sites (or simply GWAS sites). Researchers have conducted numerous GWAS studies to identify disease susceptibility sites. Whether the population specificity of these sites has an inherent connection to the population specificity of adaptive mutations is an interesting question. Research findings and theoretical analysis suggest an inherent connection, which is interpreted as a “trade‐off.” Well‐known examples include that the mutations in the *HBB* gene make some Africans resistant to *P. falciparum* malaria but also susceptible to sickle cell anemia,^[^ ^7^ ^]^ and the mutations in genes encoding hypoxia‐inducible factors (HIFs) make the carriers benefit from increased oxygen delivery but also suffer from increased blood viscosity (a contributing factor to the high incidence of stroke in Tibetans.^[^ ^8^ ^]^ These examples make us postulate that the trade‐off between adaptive evolution and disease susceptibility may be just one type of connection.^[^ ^1^, ^3^ ^]^

The challenge of identifying favored mutations lies in linkage disequilibrium (LD), which makes it difficult to differentiate favored mutations and their nearby neutral mutations.^[^ ^4^, ^9^ ^]^ Thus, LD generates a physical connection between adaptive mutations and nearby genomic sites, whatever these genomic sites are. To uncover this connection, methods and tools are needed that identify not only favored mutations but also the consequent sequence or frequency changes. Differentiating favored mutations and hitchhiking mutations is just the first step.

**Figure 1:**
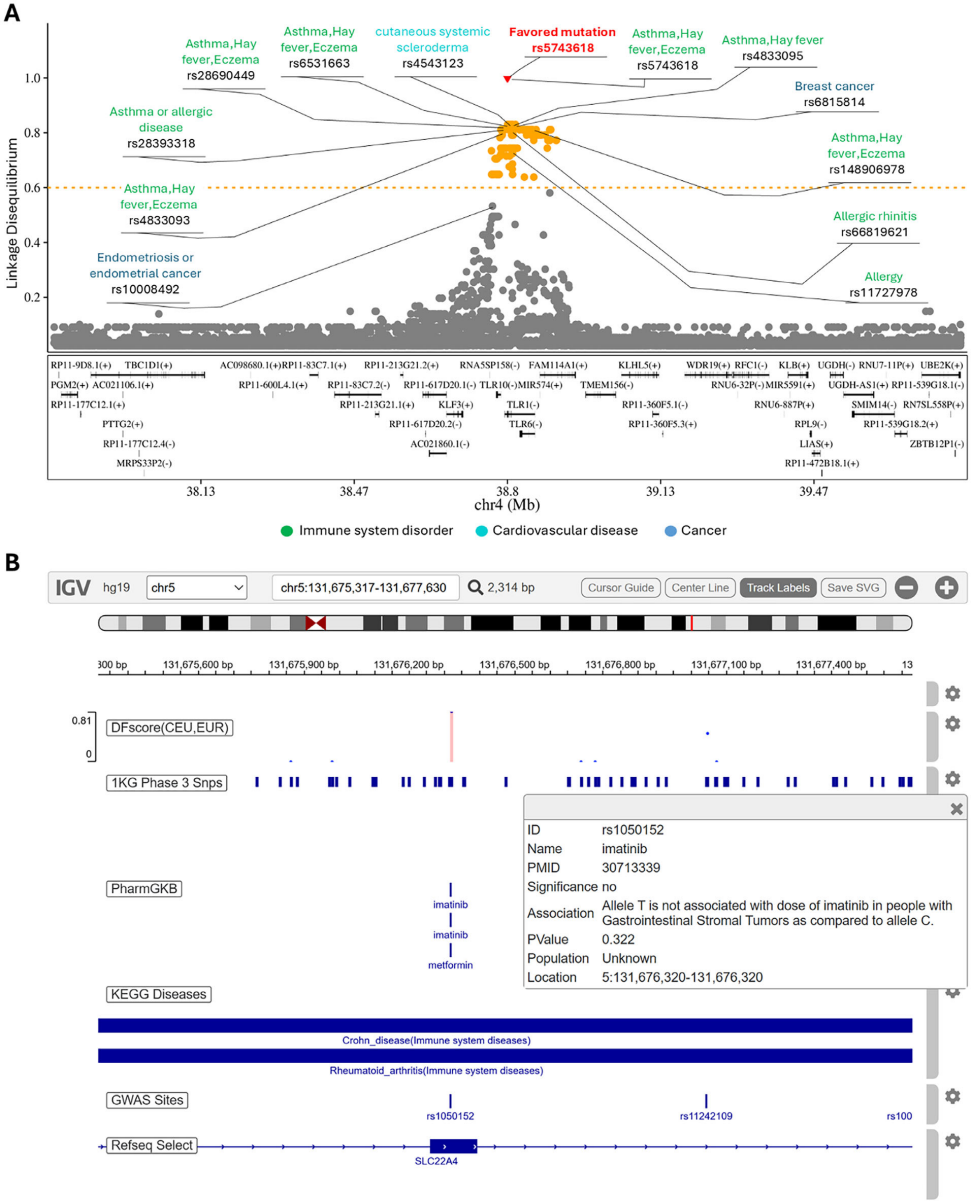
Examples of the population‐specific connections between adaptive evolution, disease susceptibility, and drug responsiveness. A) The distribution of GWAS sites (identified in CEU) at rs5743618 (a known favored mutation in CEU) in *TLR1*. The disease names and SNP IDs are shown for sites with LD > 0.5 with the favored mutation, and the dashed orange line indicates hitchhiking mutations with LD > 0.6 with the favored mutations. Red triangles, orange dots, and gray dots indicate favored, hitchhiking, and ordinary mutations, respectively (adapted from Figure 2 in^[^ ^6^ ^]^). B) The multiple connections between favored mutations, PharmGKB sites, GWAS sites, and KEGG Diseases sites in the PopTradeOff database. The inset box displays the PharmGKB record of the favored mutation rs1050152. The “1KG Phase 3 Snp”, “GWAS Sites”, and “KEGG Diseases” tracks display SNPs and GWAS sites in this genomic region, along with diseases associated with this mutation (adapted from Figure 4 in^[^ ^10^ ^]^).

Using a deep learning network (called *DeepFavored*) that integrates multiple statistical tests, we obtained several findings in previous works.^[^ ^6^, ^10^ ^]^ First, more favored and hitchhiking mutations are identified in Asian and European populations than in African populations, and most favored and hitchhiking mutations are population‐specific. Second, favored mutations are enriched in specific pathways and gene ontology (GO) terms, especially metabolism‐related ones, and the enrichment shows population‐specific features. Third, favored and hitchhiking mutations statistically significantly overlap GWAS sites associated with nervous, immune, and metabolic systems. For example, rs5743618 in TLR1 (a gene with important immune functions) is a known favored mutation in Utah residents with Northern and Western European ancestry (CEU), many immune system disorder‐associated sites identified in CEU overlap with this mutation and its hitchhiking mutations (**Figure** **1**A). Fourth, many favored and hitchhiking mutations also overlap multiple types of regulatory sites with statistical significance. The tissue distribution of the target genes of these regulatory sites suggests that favored and hitchhiking mutations influence gene expression in a tissue‐specific manner. Fifth, many favored and hitchhiking mutations overlap drug response‐related sites and/or are in exons of drug response‐related genes. For example, rs1050152 is an immune disease‐associated mutation (the associated diseases include Crohn's disease, asthma, and ulcerative colitis). The data in our database, PopTradeOff,^[^ ^10^ ^]^ indicate that it is also a favored mutation in European populations and a drug response‐related mutation that causes different populations to respond differently to drugs such as Imatinib and Ustekinumab (Figure 1B). We interpret these results as evidence of extensive and population‐specific trade‐offs between favored mutation, disease susceptibility, and drug responsiveness. This interpretation may be incomplete; instead, “everything is connected to everything else” may be more plausible.^[^ ^11^ ^]^ This commentary, from both retrospective and prospective views, addresses this topic and related issues.

## 2 Differences between Human Populations May be Greater than Assumed

Varied findings have revealed differences between human populations. In addition to favored mutations, well‐acknowledged findings also include GWAS sites and drug response‐related sites. However, several factors have been somewhat overlooked or need further investigation. The first is that all sites in selective waves may somewhat differentiate human populations; thus, accurate annotation of hitchhiking mutations is needed. The second is that, while some connections between favored mutations and disease‐associated sites are straightforward (e.g., those in genes in the GO term “insulin receptor binding” and the GWAS trait “Fasting blood glucose”), many still lack annotations. Interesting examples include the connection between the “Coffee consumption” GWAS trait and the “short‐term memory” GO term, and the connection between the favored mutations in genes in the GO term “learning or memory” and the GWAS sites of the GWAS trait “Low‐density lipoprotein cholesterol levels”. The third is that favored mutations are population‐specifically enriched in multiple GO terms,^[^ ^6^ ^]^ and the cross‐population differences in some GO terms deserve attention. Of special interest are metabolism‐related (e.g., “glucose import in response to insulin stimulus”) and nervous and sensory system‐related GO terms (e.g., “short‐term memory” and “sour taste receptor activity”). Different numbers of related favored mutations are identified in CEU, Han Chinese in Beijing (CHB) and Yoruba in Ibadan, Nigeria (YRI); whether this implies a cross‐population difference in the evolution and function of the brain is intriguing.

In previous work, we attempted to explore the forms of connection between favored mutations and GWAS sites.^[^ ^6^, ^10^ ^]^ The connection can be classified into a) overlapping of favored/hitchhiking mutations with GWAS sites, b) co‐enrichment of favored/hitchhiking mutations in genes in GO terms and genes involved in GWAS traits, and c) co‐localization of favored/hitchhiking mutations and GWAS sites at multiple types of quantitative trait loci (QTL). These QTLs include expression QTL (eQTL), methylation QTL (mQTL), histone QTL (hQTL), and splicing QTL (sQTL). Notably, favored mutation‐related eQTLs and mQTLs exist more in tissues such as the brain and skin that have evolved rapidly. These results reveal several potential mechanisms that cause the above forms. The first is the Hitchhiking of ordinary mutations. Hitchhiking mutations greatly outnumber favored mutations and thus overlap more GWAS sites. Results from several studies suggest that schizophrenia (which has many GWAS sites) has evolved and has been maintained in part as a maladaptive byproduct of recent positive selection and adaptive evolution in human beings. The second is the Primary selection of GWAS sites. Studies revealed that some sites identified by GWAS for obesity and type‐2 diabetes (T2D) have been subject to recent selection pressures in certain populations.^[^ ^12^ ^]^ Other examples mentioned above also indicate the primary selection of GWAS sites, including that the mutations in the *HBB* gene make some Africans resistant to *P. falciparum* malaria but also susceptible to sickle cell anemia^[^ ^7^ ^]^ and that the mutations in genes encoding hypoxia‐inducible factors (HIFs) make the carriers benefit from increased oxygen delivery but also suffer from increased blood viscosity.^[^ ^8^ ^]^ The third is the change in key variables during adaptive evolution. Some favored mutations may no longer be favored when new external changes occur, and some disease susceptibility sites may make the carriers no longer susceptible to the disease when new external changes occur. The changes in lifestyle, including dietary structure, provide evidence for both situations. In other words, both favored mutations and GWAS sites are conditional.

Analyses across humans and other species, as well as archaic and modern humans, also help reveal the connections. In another study, we identified human‐specific (HS) lncRNAs, predicted their DNA binding sites (DBS), and analyzed these DBS and their counterparts in modern humans (CEU, CHB, YRI), ancient humans (Altai Neanderthal, Denisovan, Vindija), and chimpanzees.^[^ ^13^ ^]^ Genes with the most polymorphic DBS from chimpanzees to modern humans include *TAS1R1* (responding to the umami taste stimulus and recognizing diverse natural and synthetic sweeteners), *INS* (decreasing blood glucose concentration), and *FN3KRP* (the encoded protein deglycates proteins whose function is deactivated by glycation, a process called non‐enzymatic oxidation caused by high sugar levels). Selection signals in many DBSs show population‐specific differences. Based on DBS sequence distances from modern humans to chimpanzees and Altai Neanderthals/Denisovans and the cutoff of 0.034 (distance per base), we divided DBSs into “old” and “young” ones. This cutoff identifies the top 20% of genes that are most likely to differentiate humans from chimpanzees. Old DBSs have a “Human‐Chimp” distance > 0.034 and a “Human‐Altai” distance = 0, indicating the mostly changed DBSs from chimpanzees to archaic humans; young DBSs have a “Human‐Altai” distance > 0.034 or “Human‐Denisovan” distance > 0.034, indicating DBSs that have undergone great changes recently. Additionally, based on the computed binding affinity, we divided DBSs into strong and weak ones (affinity > 60 and < 60, respectively). We found that a higher ratio of favored and hitchhiking mutations in the aforementioned 17 populations is in young weak DBSs than in other groups. This suggests that differentiating favored mutations in old and young functional sequences also helps reveal properties of favored mutations and GWAS sites.

## 3 Challenges of Investigating Key Variables in Adaptive Evolution Events

**Figure 2:**
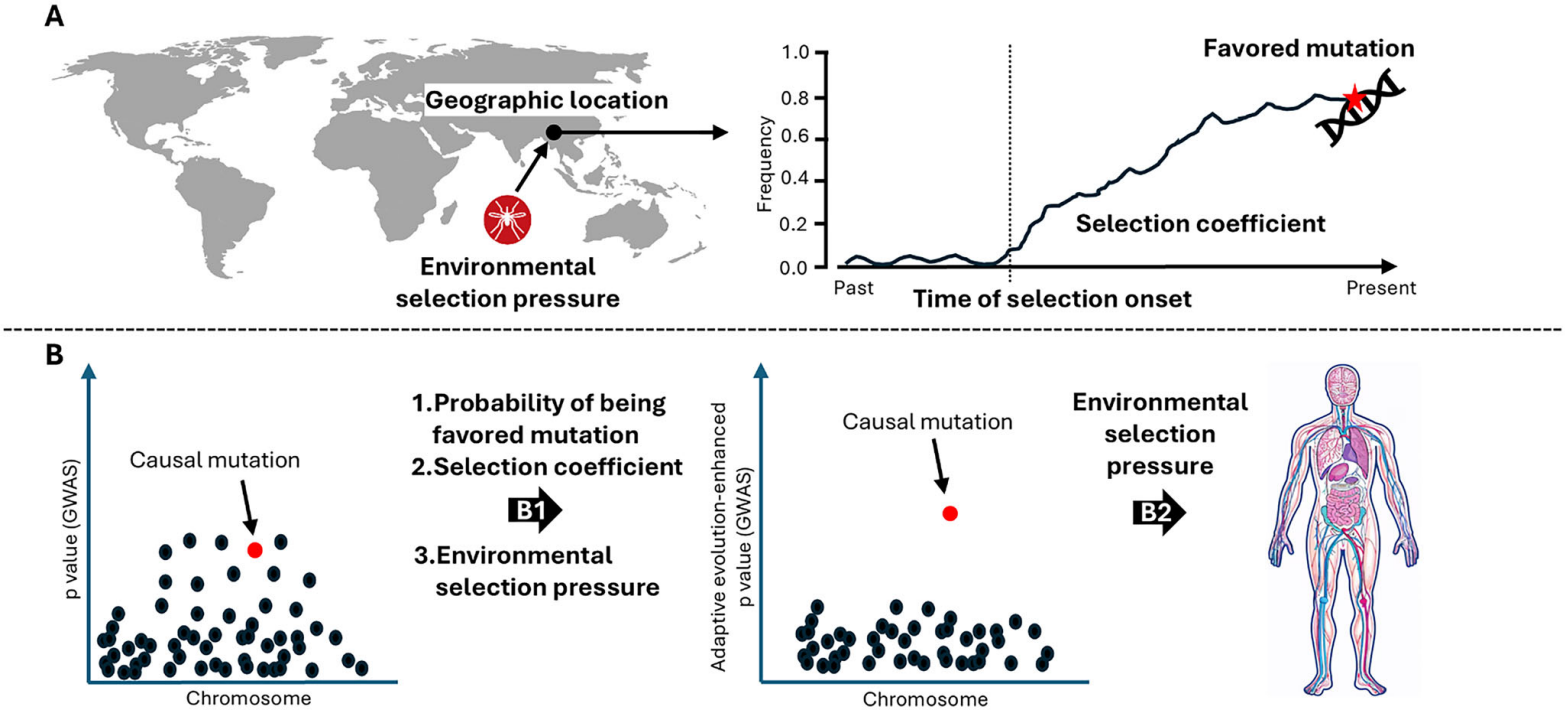
Inferring key variables in adaptive evolution events. A) Several key variables are involved in adaptive evolution events. B) Leveraging these variables helps identify causal mutations and interpret genotype‐phenotype relationships in GWAS. “B1” indicates pinpointing causal mutations based on the probability that a mutation is favored, the selection coefficient, and the environmental selection pressure. “B2” indicates that the environmental selection pressure can be very informative for investigating the phenotypic effects of favored mutations and GWAS sites.

Adaptive evolution events involve multiple variables, including the geographic location, population history, environmental selection pressure, and onset time of each event (**Figure** **2**A). The geographic locations of adaptive events are typically those of the studied populations; however, population history can be complex, as it may include recent migration or admixture events that can introduce favored mutations from distant origins. Studies have investigated several variables. Environmental selection pressures encompass various factors that may affect human physiology, including climate, altitude, pathogens, and diet.^[^ ^2^ ^]^ Although the full history of selection pressures is difficult to determine, some studies have collected and examined environmental variables that could potentially be the source of selection pressure. Specifically, Hancock et al. developed the dbCLINE database that collected 12 environmental variables covering climate, ecoregion, and diet or mode of subsistence across 61 populations worldwide.^[^ ^14^ ^]^ New progress in archaeology will identify more environmental variables.

Accurately inferring the selection strength and onset time has been a challenge. Multiple methods have been developed for this task. Nakagome et al. used Approximate Bayesian Computation to develop a method for inferring the onset time of adaptive evolution.^[^ ^15^ ^]^ Smith et al. developed a Hidden Markov Model‐based method (called startmrca) for inferring the onset time of selection.^[^ ^16^ ^]^ Hejase et al developed a method (called SIA) that combines ancestral recombination graph (ARG) and a Long Short‐Term Memory (LSTM) architecture (which is a kind of deep learning network) to infer the onset time and strength of selection.^[^ ^17^ ^]^ Recently, Vaughn and Nielsen developed a method (called CLUES2) to infer the onset time and strength of selection.^[^ ^18^ ^]^ Due to the difficulties of inferring the onset time and strength of selection, these methods face the trade‐off between sensitivity and specificity. Cross‐population differences in demographic factors, including population size, recombination rate, and mutation rate, make adaptive evolution events leave distinct genomic signatures that mark specific scenarios of adaptive evolution. To accurately and robustly infer demographic factors, new and combined use of methods is needed.

GWAS faces the challenges of identifying false‐positive results and distinguishing between causal mutations and non‐causal associations. Integrating key variables of adaptive evolution into GWAS analysis offers a solution to these challenges (Figure 2B). First, favored mutations are more likely than hitchhiking mutations to be causal mutations. Of the disease‐associated mutations identified in GWAS, those also identified as favored mutations are more likely causal mutations than those also identified as hitchhiking mutations. Second, when environmental selection pressure changes, a favored mutation may become a disease‐causing mutation, as the “mismatch diseases” hypothesis suggests.^[^ ^19^ ^]^ For example, alleles that were beneficial just thousands of years ago may become disease‐associated now after lifestyle and/or dietary structure have changed. Third, as in adaptive evolution analysis, LD is a significant contributor to high false positives in GWAS. To reduce the influence of LD, we adopted the strategy of dividing the identification of favored mutations into two subtasks. Similar strategies may be developed for GWAS. Finally, in adaptive evolution, environmental pressures directly influence phenotypes and physiological functions, and the connection between environmental pressures and the resulting changes may follow some “patterns”. Examples include that the exposure to specific pathogens specifically shapes the feature of immune responses, and the adaptation to high altitude specifically shapes the oxygen transport system. Studies focusing on these patterns may generate insightful findings, which help extend the scope of analysis and uncover mutually connected processes and outcomes of adaptive evolution. The links between the key variables in adaptive evolution events and GWAS exemplify “everything is connected to everything else”.

## 4 Probably Inherent Connections between Favored Mutations and Consequences

We made three explanations for the enrichment of hitchhiking mutations in disease‐associated mutations. These explanations, together with the “mismatch” hypothesis, suggest that a trade‐off between adaptive evolution and disease susceptibility is inherent to genome evolution.^[^ ^6^ ^]^ Rapid changes in environment or lifestyles may render alleles that were beneficial unbeneficial or disease‐associated, causing so‐called “mismatch diseases”.

On the other hand, increasing genomic sequences have been found to have regulatory functions, and considerable favored mutations are identified at sites of various QTLs, predicted DBSs of lncRNAs, and predicted DBSs of transcription factors. These findings suggest that only a portion of the connections between favored mutations and their consequent genomic changes can be classified as a trade‐off. Instead, the multiple types of key variables in adaptive evolution events suggest that other connections may exist, and multiple consequent genomic changes may be mutually connected. This property is not limited to the human genomes.

## 5 Future Directions

Existing studies for identifying favored mutations and investigating the connections between adaptive evolution, disease susceptibility, and drug responsiveness face several limitations, which reflect common challenges in this field. Here, we discuss these limitations and present potential solutions.

Supervised learning‐based methods, including DeepFavored and the alternatives SWIF(r) and CMS,^[^ ^4^, ^9^ ^]^ rely on training data simulated from population genetic models to identify favored mutations. Performance has progressively improved from CMS to SWIF(r) to DeepFavored, demonstrating that supervised learning effectively addresses this task and holds promise for developing more powerful methods. However, simulating training data that accurately represents diverse demographic histories of specific populations can be challenging, particularly for new users. Stdpopsim is a community‐maintained standard library of population genetic models.^[^ ^20^ ^]^ Integrating Stdpopsim into supervised learning methods to automate the simulation could help address this challenge. The patterns of positive selection signals are shaped by many factors (recombination rate, mutational rate, selection strength, duration of selection, etc.) and thus are diverse within and across populations, potentially limiting the generalizability of a method. The problem could be alleviated by ensuring demographic model parameters cover reasonable ranges that reflect diverse demographic scenarios during training data simulation.

DeepFavored and alternative methods, such as CMS, SWIF(r), and iSAFE,^[^ ^21^ ^]^ demonstrate limited power in detecting favored mutations at low frequencies (<0.2). The ARG, a series of genealogical trees connected through recombination events that describe joint genealogies of sampled DNA sequences along the genome,^[^ ^22^ ^]^ may offer a solution. Since low‐frequency mutations are located on recent branches in these genealogical trees, integrating ARG into method design may enable the detection of favored mutations at low frequencies.

Well‐known favored mutations are currently few; consequently, performance evaluations of DeepFavored and its alternatives on real data are limited. As more favored mutations in real data are established in the future, it is essential to incorporate them into performance evaluations.

Most existing studies have utilized mutations called with Genome Reference Consortium Human Build 37 and 38 (GRCh37 and GRCh38) references. In contrast to GRCh37 and GRCh38, recently completed Telomere‐to‐Telomere (T2T) genomes provide complete, gapless chromosome assemblies that resolve previously inaccessible repetitive regions and improve variant calling accuracy.^[^ ^23^, ^24^ ^]^ Future studies using T2T‐based mutations are expected to identify more favored mutations with higher confidence. Moreover, while previous studies discussed in this commentary used mutations from the 1000 Genome Project (phase 3),^[^ ^25^ ^]^ which covers 26 populations from 5 continental regions, newer human genetic diversity projects such as All of Us^[^ ^26^ ^]^ and NHLBI TOPMed^[^ ^27^ ^]^ provide broader geographic coverage and better representation of historically underrepresented populations and ancestry groups. Future studies leveraging these expanded projects will identify more population‐specific favored mutations.

Finally, the current disease‐ and drug‐responsiveness‐associated sites, as documented in the GWAS catalog, PharmGKB, and DrugBank databases, are insufficient and predominantly identified in European populations, thereby underestimating the connections. Fortunately, this issue has gained significant attention; more association studies are being conducted, and more GWAS sites will be identified in underrepresented populations.^[^ ^26^, ^28^, ^29^ ^]^

Future studies implementing these improvements, combined with more accurate inference of key variables in adaptive evolution events, will not only discover more instances like those in (Figure 1), but more importantly, reveal the full scale of the connections, ultimately advancing precision medicine.

## Conflict of Interest

The authors declare no conflict of interest.

## Author Contributions

J.T. and H.Z. wrote and revised the manuscript.

## Data Availability

The authors prefer not to publish the record of transparent peer review.
